# Expression of salivary immunoglobulins and their association with analgesic neuropeptide opiorphin in anorexia nervosa during adolescence

**DOI:** 10.1186/s40337-022-00637-3

**Published:** 2022-08-11

**Authors:** Elzbieta Paszynska, Amadeusz Hernik, Agnieszka Slopien, Yves Boucher, Marta Tyszkiewicz-Nwafor, Magdalena Roszak, Karolina Bilska, Monika Dmitrzak-Weglarz

**Affiliations:** 1 Department of Integrated Dentistry, Poznan University of Medical Sciences (PUMS), Bukowska St. 70, 60-812 Poznan, Poland; 2 Department of Child and Adolescent Psychiatry, PUMS, Poznan, Poland; 3 Université de Paris, LabNOF, 75013 Paris, France; 4 Groupe Hospitalier Pitié Salpêtrière, Paris, France; 5 Department of Computer Science and Statistics, PUMS, Poznan, Poland; 6 Department of Psychiatric Genetics, Department of Psychiatry, PUMS, Poznan, Poland

**Keywords:** Anorexia nervosa, Saliva, Immunoglobulins, Opiorphin, Oral hygiene

## Abstract

**Background:**

Patients who suffer from anorexia nervosa (AN) are characterized by exceedingly lower body weight, micro- and macro-nutrient deficiencies, and hyposalivation as compared to healthy subjects. In addition, AN may predispose to difficulties in oral health maintenance. However, little is known about the relationship between stress-dependent salivary neuro/immunopeptidergic biomarkers such as opiorphin and immunoglobulins (Ig) and AN.The aim of this case–control study was to evaluate salivary opiorphin and immunoglobulins in female children and adolescents diagnosed with AN compared to healthy controls.

**Methods:**

Adolescent patients with clinically-confirmed severe restrictive subtype AN (Body Mass Index BMI < 15 kg/m^2^, mean age 15.0 ± 1.8, n = 83) were examined in the first week of hospital admission and compared to healthy matched controls (n = 79). Measurements of salivary opiorphin, IgA, IgG, IgM (ELISA technique), and oral hygiene levels (Plaque Control Record index—PCR) were performed.

**Results:**

In the AN group, a significantly higher concentration of opiorphin was evidenced (3.1 ± 4.1 ng/ml) compared to the control group (1.1 ± 1.2 ng/ml), (p < 0.001), contrary to IgM, which was significantly lower (311.0 ± 185.3 ng/ml) than in the control group (421.2 ± 168.1 ng/ml), (p < 0.001). There were no significant differences in the levels of IgA and IgG, despite a higher concentration of IgA in the AN group vs. controls (p = 0.14). Spearman analysis revealed a correlation between opiorphin and age (p < 0.05), but also with all immunoglobulins IgA, IgG, IgM (p = 0.006, p < 0.001, p < 0.001). Similarly a correlation was found between PCR index and immunoglobulins IgG, IgM (respectively p = 0.028, p < 0.001), and between body mass, BMI, IBW% and IgA, IgM (all p < 0.05).

**Conclusions:**

In the acute phase of AN, salivary changes in opiorphin and immunoglobulins related to dental plaque suggest an essential role in oral health balance. Changes related to AN may affect the anti-inflammatory and analgesic components of saliva and suggest their use as neurobiological markers in severe malnutrition.

**Supplementary Information:**

The online version contains supplementary material available at 10.1186/s40337-022-00637-3.

## Plain English summary

The orofacial region is affected by various inflammatory and autoimmune conditions, which might translate into general and regional changes. Saliva is one of the biological fluids where biomarkers of the aforementioned conditions, including sensitivity to pain, could be detected. It is also noteworthy that anorexia nervosa (AN) and malnutrition may change the saliva's analgesic or immune content, but this scientific area is still not satisfactorily explored. The present work related to analgesic (opiorphin peptide) and anti-infectious agents (immunoglobulins Ig A, IgG, IgM) in said body fluid among 83 adolescent patients with severe AN compared to 79 healthy controls. In addition, oral hygiene levels were assessed in the oral cavity via the Plaque Control Record index (PCR). In the AN group, the concentration of opiorphin was significantly higher as compared to the control group, in contrast to IgM, which was significantly lower than in the control group. There were no notable differences in the levels of IgA and IgG between groups, despite a slightly higher concentrations of IgA in the AN group. A correlation was found between opiorphin and all immunoglobulins. A similar correlation was found between PCR index and all immunoglobulins.The present work shows how analgesic opiorphin depends on the oral inflammatory status. This might be a direction for further investigating the immune alterations in patients with AN-related malnutrition. Inclusion of AN patients in intensive oral hygiene care may also be considered.

**Supplementary Information:**

The online version contains supplementary material available at 10.1186/s40337-022-00637-3.

## Background

The onset of anorexia nervosa (AN), a type of eating disorder (ED), is often observed in childhood, from the age of 12-years-old (y.o.) or younger [1] (with a lifetime prevalence under 5%) [2]. This period is crucial for oral homeostasis notably of deciduous and permanent teeth, periodontal tissues, oral mucosa, and oral fluids [3]. Few investigations have focused on oral health-related aspects of adolescents suffering from AN, during the first period of their eating disorder, i.e. under the age of 18 y.o. [4–6]. In AN, permanent body starvation and nutritional deficiencies, combined with low salivary gland production, impair orofacial functions [4–7]. Anorexic behavior reduces the number of meals/chewing activity in the oral cavity, decreases salivary output, and alters saliva composition and sensory perceptions [1, 8, 9]. Recent research on animal models has demonstrated that chewing could help attenuate the stress-induced neurophysiological response [10, 11]. This effect might be mediated, at least in part, by saliva that besides its lubricative properties helping food bolus transformation, is known to be part of a neuroendocrine axis [12]. Changes related to the AN disease may affect saliva's anti-inflammatory and analgesic components, such as immunoglobulins A, G, M, and other compounds like opiorphin [13].

Opiorphin, the human peptide analog of rat sialorphin, is an inhibitor of neutral endopeptidase and a member of a family of enzyme-degrading peptides, thus increasing their bioavailability. It is found in body fluids such as saliva, blood serum, urine, milk, semen, and tears [14, 15] and shows analgesic properties in several animal models with a reported six-fold stronger analgesic potency than morphine [16, 17]. In addition, Rougeot et al. (2010) [18] showed that opiorphin, unlike opioid drugs, causes fewer side effects, does not induce drug tolerance, and is not addictive [19, 20]. Singh et al. (2018) [21] found a lower analgesic effect of opiorphin at the supraspinal level than morphine, probably related to its rapid degradation or its limited ability to cross the blood–brain barrier estimated at 3% [22].However, its central action might exacerbate an antidepressant effect due to the more prolonged accessibility of enkephalins to µ- and δ-opioid receptors [19, 23, 24].

IgA, the principal immunoglobulin found in saliva, prevents the adhesion of microorganisms to oral mucosal epithelial cells by binding and agglutination. IgG and IgM act as opsonins, and their antibacterial properties are expressed by binding to foreign microorganisms or cells, making them more susceptible to phagocytosis. Altogether, they have a protective effect against human pathogens, either directly or indirectly, due to their role in stress [25–27]. Increasing evidence suggests that stress can decrease immunoglobulin concentrations in saliva [28–33]. Patients with neuroendocrine alterations, including ED, are susceptible to immune system alterations that affect salivary immunoglobulins levels, either through the autonomic nervous system (ANS) or hypothalamic-adrenal–pituitary axis (HPA). However, limited data are available in the literature regarding their contribution to secretion or depletion of recently-discovered opiorphin and immunoglobulins regarding the acute phase of AN in adolescents. Therefore, this case–control study aimed to explore the relationship between salivary biomarkers of analgesia and immunity in AN adolescent patients as compared to healthy controls.

The main objective was to compare salivary opiorphin, IgM, IgG, and IgA levels assessed using ELISA tests in adolescents diagnosed with AN to healthy dental patients (controls).

Secondary objectives were to investigate putative relationships between salivary biomarkers and individual parameters: oral hygiene, disease duration, weight, Body Mass Index (BMI), and Ideal Body Weight (IBW).

## Methods

### Study participants

This case–control study was conducted in adherence to Good Clinical Practice guidelines and the recommendations of the Declaration of Helsinki after approval by the Bioethics Committee of Poznan University of Medical Sciences (Resolution No. 489/19). An explanation of the purpose and principles of the study was conveyed to all 162 children, who gave their informed written consent to participate in this study. Expression of their personal beliefs was individually searched before the examination. Additionally, a parent or legal guardian's approval was needed for inclusion in the study. A lack of acceptance from patients, parents, or legal guardians excluded from participation in the study. Informed consent was obtained from parents/legally authorized representatives for all participants.

Anthropometric data were collected, such as age, sex, body height, weight and calculated body mass index (BMI) as a ratio of body weight [kg] to height [m]^2^. The percentage of ideal body weight (%IBW) was calculated as a ratio of actual to ideal body weight (IBW) × 100%, where IBW (kg) = height (cm) − 100 − ([(height (cm) − 150)]/2) according to Lorentz’s formula [34].

Based on inclusion and exclusion criteria, the subjects were assigned to anorexic (AN) and control groups (Table 1).

**Table 1:** Inclusion and exclusion criteria for both study groups (with respect to ICD-10, DSM-V)

Criteria for Inclusion into the Study Group (AN)	Criteria for Inclusion into the Control Group	Criteria for Exclusion from Study and Control Groups
Female patients aged 12–18	Female patients aged 12–18	Children/adolescents with disorders of the central nervous system (e.g. epilepsy, serious injuries, and CNS infections)
Children/adolescents with diagnosed AN restrictive subtype as per ICD-10 and DSM-V diagnostic criteria (diagnosis confirmed by two independent psychiatrists)	Lack of mental disorders	Co-existing: schizophrenia, bipolar affective disorder, any serious somatic disorder
Clinically significant AN symptoms lasting over six months	No past or current ED symptoms	Chronic somatic diseases
BMI < 17 kg/m^2^	BMI 17–24 kg/m^2^	BMI > 25 kg/m^2^
Children/adolescents without hereditary mental disorders (first-degree relatives)	Children/adolescents without any hereditary mental disorder (first-degree relatives)	Persistent pharmacotherapy Hormonotherapy Contraception Pregnancy Dietary supplements
Patient and parent or legal guardian approval	Patient and parent or legal guardian approval	Lack of acceptance from patients, parents, or legal guardians
		Smoking
		Professional scaling Orthodontic treatment Antibiotic therapy Anti-inflammatory drugs 3 months before a dental examination

*AN* anorexia nervosa, *ED* eating disorders, *ICD-10* International Statistical Classification of Diseases and Related Health Problems (10th edition), *DSM-V* Diagnostic and Statistical Manual of Mental Disorders (5th ed.), *CNS* Central Nervous System; *BMI* Body Mass Index

*The AN group* included eighty-three female adolescents in the acute phase of AN referred to the same public Department of Child and Adolescent Psychiatry. Diagnosis of the restrictive subtype of AN was confirmed following a semi-structured interview by a child and adolescent psychiatrist according to ICD-10 (code F50.1) [35] and DSM-5 (code 307.1) criteria [36]. All patients in this group had similar clinical characteristics (restrictive type) and menstrual status (secondary amenorrhea). Some patients had depressive and obsessive–compulsive symptoms, but less than 20% required pharmacotherapy 2 or 3 week after admission) [8, 37]. The clinical examination and collection of salivary samples were performed during the phase of severe symptoms (BMI < 15 kg/m^2^) during first week of the patients’ hospitalization (1^st^). All patients had collected a medical history to check that eating symptoms lasted less than twelve months. To achieve homogeneity among the participants, patients of the bulimic type and those suffering from any other somatic disorder were excluded from the study [35, 36]. Other exclusion criteria were: chronic somatic diseases or other mental/neurodevelopmental disorders (any primary disease implicating an eating disorder), hereditary disorders (first-degree relatives), pharmacotherapy, hormonotherapy, pregnancy, contraception, dietary supplements or smoking.

*The control group* consisted of healthy teenagers recruited among patients attending routine dental care in the University dental clinic matched in age (12–18 years) and sex in respect to the studied AN group in the same period. Patients attending urgent, orthodontic, or non-routine dental treatment were excluded from the study. Importantly, control subjects did not report any types of eating disorders in the past. The following exclusion criteria in the control group were as follows: permanent somatic diseases, mental or neurodevelopmental disorders, hereditary disorders (first-degree relatives), pregnancy or breastfeeding, hormonal contraception, pharmacotherapy, endocrine therapy, and dietary supplements. Children whose parents were dental professionals or dental students were excluded from the study, as well as subjects who underwent any general or oral treatment capable of altering salivary composition.

**Fig. 1:**
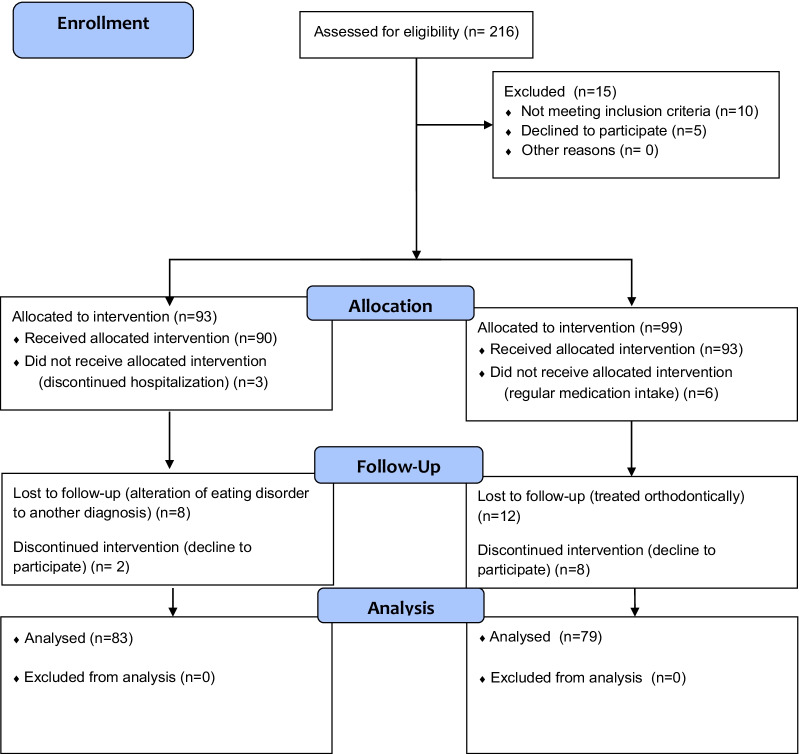
Flow chart of the study

The study flow chart is indicated in Fig. 1.

### Clinical dental examination and salivary collection

Special attention was paid to standardizing data collection. Participants from both groups were referred to the same dental office between 9:00 and 10:00 a.m. for clinical dental examination and saliva sampling, performed by the same qualified dentists (EP, AH). The examiners were trained at the start of the study and assessed for inter-examiner reliability; the oral examination parameters were acceptable if the ICC values and Cohen's Kappa coefficients were ≥ 0.9 (p < 0.001) [38]. Oral hygiene control was evaluated using the dichotomized Plaque Control Record index (PCR) [39]. The Plaque Control Record provides a simple, sequential index of plaque retention on the mesial (M), distal (D), facial (F), and lingual (L) tooth surfaces. Dental plaque was recorded using a manual graded periodontal WHO type probe (LM-Instruments, LM8 5050 probe, Osakeyhtiö, Parainen, Finland). The probe consisted of a 0.5 mm ball at the tip and had millimeter (mm) graduations at 3.5, 8.5, 11.5 mm and color-coding from 3.5 to 5.5 mm. The proportion of surfaces (%) with a dental plaque was calculated for each patient as percentage of sites [40].

Unstimulated whole saliva was collected according to previously-described methodology [4, 41]. The patients were recommended not to eat one hour prior to the examination and to forgo any medical or oral hygiene procedures before the visit. Saliva collection of hospitalized patients was conducted after their regular breakfast at 8:00 a.m. For body fluid collection, subjects were asked to spit a total average of 2 ml unstimulated saliva into a sterile container for 15 min, spitted in two periods of 7′30, each collected in a separate flask. During the examination children were asked to focus on spitting, limit other activities, and were leaned forward whilst in a seated position. Immediately after collection, the samples were centrifuged; the separated supernatant was first frozen at −20 °C and then at −80 °C until further biochemical processing.

### Opiorphin measurement

The quantification of opiorphin in saliva was performed using a commercial enzyme immunoassay kit (ELISA test for measuring human opiorphin cat. no. EH1927, FineTest, Wuhan, Hubei, China) according to the manufacturer's instructions. The measuring range of the kit was 0.156–10 ng/ml, with a sensitivity of 0.094 ng/ml. The intra-platelet coefficient of variation was < 8%, with an inter-platelet variation coefficient of < 10%.

*Secretory IgA, IgG, IgM measurements in human saliva* were performed using ELISA kits (ELISA Kit, DEMEDITEC, no. DEXK276, Demeditec Diagnostics GmbH, Kiel, Germany; ELISA Kit, AMSBIO no.0544 h, EIAab, Wuhan, China; ELISA Kit, AMSBIO, no. E0543h, EIAab, Wuhan, China).

*Salivary IgA* concentrations were quantitatively determined by the ELISA method using the ELISA Kit for In Vitro Diagnostic (IVD). The standard curve ranged from 0 to 400 µg/ml, the intra- and inter-assay variability coefficient were assessed to be below 5%, and the standard curve was statistically significant (r^2^ = 0.995, p < 0.001) [27, 42].

*Salivary IgG* concentrations were quantitatively determined by the ELISA method. The standard curve ranged from 0 to 5000 pg/ml, the intra-assay variability coefficient was < 4.4% and inter-assay variability was < 7.8%, respectively (r^2^ = 0.999, p < 0.001) [43, 44].

*Salivary IgM* concentrations were quantitatively determined by the ELISA method. The standard curve ranged from 0 to 500 ng/ml, the intra-assay variability coefficient was < 6.2% and inter-assay variability was < 9.1%, respectively (r^2^ = 0.986, p < 0.001) [45].

All ELISA tests were performed according to the manufacturer's instructions, without any modification. All samples and standards were run in duplicates, and the mean value of the two assays was used for statistical evaluation. Optical density was read with a spectrophotometric plate reader (Asys UVM 340 Microplate Reader from Biochrom Ltd., Cambridge, UK) at a wavelength of 450 nm ± 10 nm. A four parameter algorithm (4 parameter logistic) was used to measure the concentration in the tested samples.

All tests were performed by an investigator blind to the clinical data and status of the samples (group allocation).

### Statistical analysis

The analyzed data were expressed as mean ± standard deviation, median, minimum and maximum values, interquartile range, or percentage, as appropriate. Normality of distribution was tested using the Shapiro–Wilk test (interval scale). Two unpaired groups were compared using the Mann–Whitney U-test (data were not normally distributed or ordinal data). The relationship between variables was analyzed with Spearman's rank correlation coefficient (when data were not normally distributed or ordinal data). Categorical data were analyzed with the χ^2^ test or the Fisher-Freeman-Halton test (or contingency table large than 2 × 2 with any expected values was less or equal to 5). Statistical analyses were performed with STATISTICA 13.0 (StatSoft Inc., Tulsa, USA) or StatXact 11.0 (Cytel Inc., Waltham, Massachusetts, USA). Multivariate analysis as logistic regression (backward, forward) was also carried out to determine risk factors significantly affecting the AN group. The odds ratio and 95% confidence intervals were set for the indicated variables. This way, the answer to the question was which of the independent variables significantly influenced the AN group. Therefore, a relationship was sought between the probability of disease occurrence and the group of independent variables. The parameters taken for analysis were selected following previous research and the observations found in the literature. The group of variables included in the logistic regression were: BMI, duration of AN illness, opiorphin levels, immunoglobulins IgA, IgG, IgM levels and PCR. Logistic regression calculations and intra-examiner calibration results (ICC) and Cohen's Kappa coefficient were performed in a statistical package MedCalc v. 19.5.1 (MedCalc Software, Ostend, Belgium). All results were considered significant at p < 0.05.

#### Sample size

Considering the size of the target AN population, the sample size was based on European data estimating between 8–13 AN cases for 100,000 adolescent females. Similarly, the nationwide AN frequency is estimated between 0.8–1.8% of girls under 18 y.o. [46, 47]. It was calculated using Cochran's formula [48] that at least 36–59% of the target AN eligible population individuals should be surveyed to reach a margin level of 2% at the confidence level of 95%.

The reporting of the study was made according to the Strengthening the Reporting of the Observational Studies in Epidemiology (STROBE) guidelines (Additional file 1).

## Results

### Sample

The final sample consisted of 162 participants (83 AN patients and 79 control subjects) (Fig. 1). The age of the participants ranged from 12 to 18 y.o. The subjects from both groups were Caucasians with high school level education. There were no differences between AN and control subjects regarding religion (primarily Catholic), domestic pets (mainly dogs), or education level (school enrolled children). The mean age of AN patients was 15.0 ± 1.7 years, and the duration of the eating disorder lasted on average 10.8 ± 6.4 months. The mean age of controls was 15.0 ± 1.9 years with no statistically significant difference with AN patients (p > 0.05, Mann–Whitney U test). Bodyweight, height, BMI, and IBW were statistically different between patients and controls (p < 0.001, Welch test). The examined AN patients had on average 27.2% lower BMI than healthy controls.

### Dental plaque examination

The mean percentage of sites with plaque deposits was significantly higher in the AN group than in controls (42.6 ± 24.5 vs. 10.5 ± 14.3, p < 0.001).

**Fig. 2:**
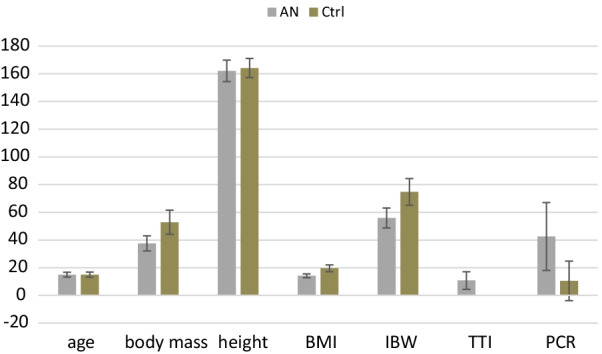
Summary of age and anthropometric parameters in AN (n = 83) and control (n = 79) groups and duration of disease for the AN group. Results are expressed as mean ± SD. Significant values from p < 0.05, ns- statistically non-significant, n- number of patients, SD- standard deviation. Statistical tests used: Mann–Whitney U test, t-test, or Welch test. BMI- Body Mass Index [kg/m^2^], IBW- % of Ideal Body Weight (fraction), TTI- total duration of illness [months], PCR- Plaque Control Record Index, AN-anorexia nervosa group, Ctrl-control group

The main characteristics of the sample are summarized in Fig. 2 (Additional file 2: Table S1).

### Salivary biomarkers

**Fig. 3:**
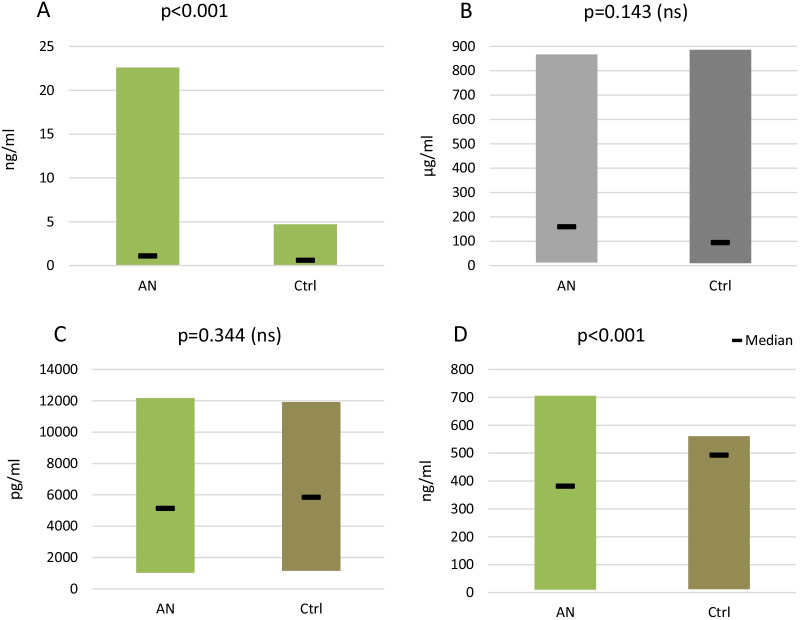
Concentration of opiorphin (**A**), immunoglobulins IgA (**B**), IgG (**C**), and IgM (**D**) in unstimulated whole saliva. The results are expressed as Median and min–max ranges. Significant values from p ≤ 0.05, p ≤ 0.01, p ≤ 0.001, ns—statistically non-significant, n—number of patients, AN-anorexia nervosa group, Ctrl-control group. Statistical tests used: Mann–Whitney U test, t-test, or Welch test

The main characteristics of the salivary samples are summarized in Fig. 3. Opiorphin levels in unstimulated total saliva were significantly higher in the AN group than in the control group (p < 0.001, Mann–Whitney U test), leading to the rejection of the null hypothesis, as differences between the two groups were evidenced.

Analysis of the salivary immune biomarkers revealed a significant reduction in IgM (p < 0.001) levels in the AN group as compared to controls. No differences in IgA (p = 0.143) and IgG (p = 0.344) levels were detected between AN and control groups. However, the median IgA level was higher than that of the controls. No differences in the IgA and IgG salivary content were identified.

AN-patients with anorexia nervosa, Ctrl- healthy controls. The results are expressed as median and min–max ranges. Significant values from p ≤ 0.05, p ≤ 0.01, p ≤ 0.001, ns- statistically non-significant. Statistical tests used: Mann–Whitney U test, t-test, or Welch test (Additional file 2: Table S2).

### Correlations

Spearman's analysis of the AN group results revealed a correlation between opiorphin and all immunoglobulin levels: IgA, IgG, IgM (respectively p = 0.006, p < 0.001, p < 0.001). A similar correlation between PCR index and immunoglobulins IgG and IgM (respectively p = 0.028, p < 0.001) was also evidenced. Analysis of anthropometric parameters "body mass", "BMI", and "IBW%" showed a significant correlation with IgA and IgM levels (p < 0.05), as was found between age and opiorphin (p < 0.05).

In the control group, correlations were also observed between the above-mentioned variables measured in saliva. Opiorphin was negatively correlated with IgM (p < 0.001) and positively with PCR index (p = 0.003). PCR index showed a significant correlation with IgG and IgM levels (respectively p = 0.024, p < 0.001).

**Fig. 4:**
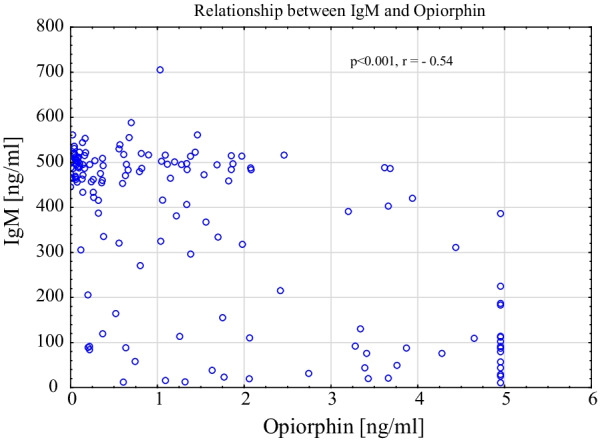
Scatterplot of salivary IgM and opiorphin relationship in AN group

This data set is summarized in Table 2 and the correlation between opiorphin and IgM illustrated in Fig. 4.

**Table 2:** Spearman's rank-order correlations between studied parameters in total, AN,and control groups

Variables	Spearman's rank-order correlations					
	Total n = 162		AN group n = 83		Control n = 79	
	r_s_	p value	r_s_	p value	r_s_	p value
Opiorphin vs. IgA	ns	0.316	0.30	0.006	ns	0.080
Opiorphin vs. IgG	−0.39	< 0.001	−0.54	< 0.001	ns	0.294
Opiorphin vs. IgM	−0.53	< 0.001	−0.54	< 0.001	−0.44	< 0.001
Opiorphin vs. PCR	0.24	0.003	ns	0.509	0.34	0.003
IgA vs. IgG	ns	0.336	ns	0.089	ns	0.560
IgA vs. IgM	−0.28	< 0.001	−0.48	< 0.001	ns	0.529
PCR vs. IgA	0.21	0.007	0.24	0.028	ns	0.229
PCR vs. IgG	0.19	0.017	0.43	< 0.001	0,25	0.024
PCR vs. IgM	−0.45	< 0.001	ns	0.379	−0.42	< 0.001
body mass vs. IgA	ns	0.327	0.28	0.011	ns	0.077
body mass vs. IgM	0.30	< 0.001	−0.26	0.020	ns	0.555
BMI vs. IgA	ns	0.564	0.34	0.002	ns	0.222
BMI vs. IgM	0.30	< 0.001	−0.28	0.011	ns	0.57
IBW vs. IgA	ns	0.683	0.27	0.014	ns	0.991
IBW vs. IgM	0.28	< 0.001	ns	0.104	ns	0.886
age vs. opiorphin	ns	0.830	0.22	0.047	ns	0.091

*n* number of patients, *ns* statistically non-significant, *PCR* Plaque Control Record Index, *BMI* Body Mass Index, *IBW* Ideal Body Weight index, *vs.* versus

*A logistic regression* model (backward, forward) was also carried out, indicating three variables: BMI, IgM, and PCR, which were statistically significant at p < 0.05. The odds ratio (OR) of the BMI parameter was 0.03, IgM 0.99 and PCR 1.08 (> 1).

## Discussion

**Fig. 5:**
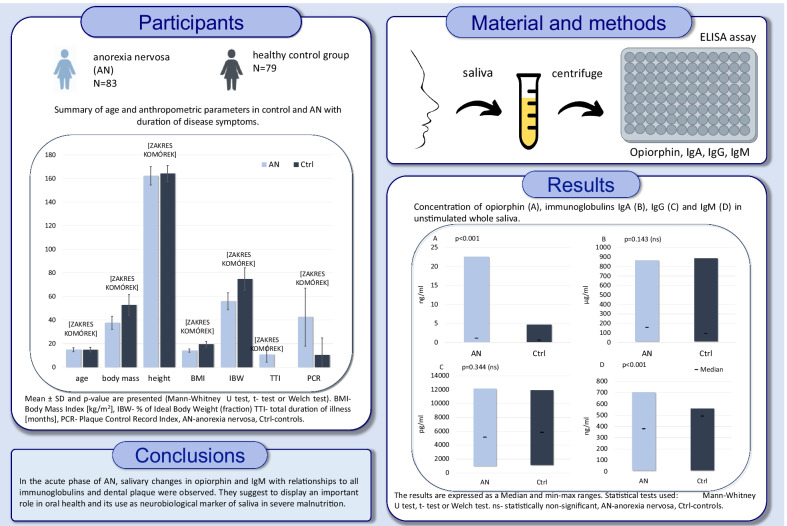
Graphical abstract of the study

The main results of this study are differences in salivary opiorphin and IgM levels between anorexia nervosa patients (AN group) and healthy controls (Fig. 5). No significant differences were found regarding the IgA and IgG levels between the two groups. AN patients presented less efficient dental plaque control than control subjects.

Opiorphin is a biomolecule of interest in pain, stress, and eating behaviors which are critical features of AN [16–24, 49]. Numerous studies have confirmed the activation of the stress HPA axis in AN patients [32, 50] and recent clinical trials suggest that mental stress increases the secretion of opiorphin [51–54]. For example, Ozdogan et al. (2017) found increased opiorphin levels in adult patients experiencing different types of tooth pain [51].The same authors found significantly higher opiorphin concentration in the tears of patients with corneal trauma compared to healthy individuals. They concluded that opiorphin release occurred primarily as a pain control mechanism, although no additional stress biomarkers were measured [52]. Other studies indicate that opiorphin's level might correlate with the levels of oral pain and stress [14, 53–56]. Recently a cohort study of 503 healthy schoolchildren where opiorphin levels were higher and correlated to prior stress exposure [49] suggested the use of opiorphin to evaluate stress-related changes in children [49].

AN is characterized by altered food intake and chronic stress. Interestingly Chen et al. found that opiorphin suppressed food intake in starved animals but not in controls animals [57]. They suggested that the anorexic effect of opiorphin was mediated by the opioid system and endogenous angiotensin, protected from enzymatic degradation by the peptide-degrading enzymes NEP or APN. It is important to underline that no additional biomarkers of stress were measured in the study of Chen et al. [57]. In our previous study, opiorphin levels were not significantly different for AN patients and controls subjects. However, a positive correlation was found between the duration of the disease and the score of anxiety measured by the Beck Depression Inventory test (BDI) and salivary cortisol concentration [58]. This may support an association between opioid and serotonergic systems involved in mood and anxiety disturbances [59].

In the current study, we have examined a larger group of AN patients and observed a high variability in opiorphin levels. One of the reasons may be related to hyposalivation in malnourished patients [4, 6]. Systematic reviews and meta-analyses have showed a reduced salivary flow in eating disorders. However, in the literature, there is less information regarding the risk of psychotropic side-effects, nutritional deficiency, acidic diet, or mood and anxiety disturbances [60]. According to previous studies, all the above-mentioned circumstances could cause an extended variability in the results and differences [58, 61].

The pathogenesis of AN may involve chronic mental stress resulting in activation of the HPA axis without autonomic nervous system (ANS) or immune dysregulations [32, 50, 60–67]. If monitored in parallel with the immune system, the assessment of opiorphin might then be a diagnostic marker of stress or an indicator of recovery. In the present study, the immune biomarkers IgM were reduced. The negative correlations of opiorphin with IgG and IgM suggest that stress and low nutritional status affect the immune system, with measurable changes in saliva biomarkers. Many publications point to the immune system malfunctioning in AN subjects [61, 68], with immune disturbances contributing to appetite dysregulation [68–70]. The immunoglobulins' production may depend on the bacterial flora of the gastrointestinal tract, react with hormones and neuropeptides regulating appetite and satiety, and play a vital role in the etiology of eating disorders [71–75]. In a recent study, it was suggested that immunoglobulins may play a role in feeding and nutrition. Specifically, their modulation may affect melanocortin four receptor signaling in obesity and eating disorders [75]. A comment on IgM-specific decrease might be interesting since IgM reflects a non-specific immune response. Salivary immunoglobulin activity may be masked because immune response operates on multiple levels, with compensatory mechanisms coming into play [76]. Thus, IgM is increased when IgA is not quite plentiful, which suggests an inverse relationship between salivary IgA and IgM activities [77]. We can speculate that under severe malnutrition fluctuations of IgA levels may alter IgM levels. Exploring the aforementioned relationships in future research might be fruitful.

Much attention has been paid to the effects of physical activity on salivary antibody levels. These levels may be related to the amount of physical activity aimed at reducing body weight in AN [78]. Short, intense training sessions increase IgA levels, while robust efforts lasting more than two hours decrease IgA levels [79, 80]. In the present study, no distinct increase in the IgA levels was found; however a significant correlation between IgM and dental plaque levels was observed. We can assume that obtained outcomes need to be studied in longitudinal time frame including additional factors in the context of defense mechanisms regulating body weight in the course of AN.

The present study’s results indicate that plaque deposits among patients with AN was alarmingly high and correlated with opiorphin and all immunoglobulin levels. This outcome is consistent with a study comprising older AN patients with a longer duration of disease, showing that the quantity of dental plaque was very high [81]. Based on our observations and recent literature, it seems that poor motivation to maintain good oral hygiene may also be influenced by adverse life events, related apathy, depressed mood, psychomotor drive, and suicidal tendencies [9, 58, 67, 81–83]. Conversely, the dental examination conducted at the beginning of the admission to the hospital might ameliorate the oral hygiene regime. It has to be underlined that the hospital stay is a challenging period for AN patients. They have to adapt to medical protocols, which can cause considerable stress. For example, ED patients have to eat under a nurse's supervision during hospitalization. Thus, patients cannot perform oral hygiene procedures immediately after eating. It should also be noticed that sugar consumption is favored to normalize weight, despite its adverse effects on oral health.

In summary, a strength of the study is the matching of the groups by age and sex, which presented very similar demographic features. A limitation here was its relatively small sample size, representing only AN female population seeking psychiatric treatment. The subjects in this study were all females due to hospital recruitment during the period considered. To the best of our knowledge, there is no comparative study concerning salivary results among AN males, probably because of the high female to male (10:1) ratio in AN [81]. A notable limitation was also a lack of data regarding earlier oral hygiene levels in both AN and control groups. This comparison could bring additional insights for all children suffering from ED. Longer follow-up periods may be suggested to evaluate the clinical approach to oral health among AN patients.

Moreover, the study was also refined by the case–control design and the assessment of oral hygiene indicators based on visual criteria when examining the oral cavity. Another limitation relates to the methods of salivary collection. The levels of the antinociceptive and inflammatory biomarkers were assayed only in the unstimulated saliva, which might be different from those of stimulated saliva. Finally, the use of additional instruments for evaluating the psychic stress aspects of adolescent individuals would have brought further information as would have the measurement of other inflammatory mediators.

## Conclusions

Differences in salivary biomarkers related to immunity and stress, i.e., opiorphin and IgM, were identified in AN patients compared to controls. Patients from the AN group had poor oral hygiene, which correlated to salivary biomarkers. Although the present data showed variability in results, they suggest that salivary opiorphin, immunoglobulins IgA, IgG, and IgM are linked to dental plaque accumulation in adolescents diagnosed with severe AN. Of particular interest is the finding that opiorphin productions seem to reflect immune conditions and inflammatory status. Exploring the nature of this relationship might be interesting and still relevant, especially in AN patients presenting severe malnutrition.

## Supplementary Information

**Additional file 1.** STROBE (Strengthening The Reporting of OBservational Studies in Epidemiology) Checklist for the study.

**Additional file 2. Supplementary table S1**. Summary of age and anthropometric parameters in AN (n=83) and Ctrl (n=79) groups with duration of disease (in the AN group). **Supplementary table S2**. Concentration of opiorphin, immunoglobulins IgA, IgG and IgM in unstimulated whole saliva.

## Data Availability

Data associated with the paper are not publicly accessible but are available from the corresponding author upon reasonable request.
