# Supplementary material for: Expression of salivary immunoglobulins and their association with analgesic neuropeptide opiorphin in anorexia nervosa during adolescence
Source: J Eat Disord. 2022 Aug 11;10:118. doi: 10.1186/s40337-022-00637-3 (PMC9367138; doi:10.1186/s40337-022-00637-3)
Supplement: Supplementary file 2 — Additional file 2. Supplementary table S1. Summary of age and anthropometric parameters in AN (n=83) and Ctrl (n=79) groups with duration of disease (in the AN group). Supplementary table S2. Concentration of opiorphin, immunoglobulins IgA, IgG and IgM in unstimulated whole saliva. [file 40337_2022_637_MOESM2_ESM.docx]

Supplementary table S1. Summary of age and anthropometric parameters in AN (n=83) and Ctrl (n=79) groups with duration of disease (in the AN group).

|  | | **median** | min-max range | p-value |
| --- | --- | --- | --- | --- |
| age  [years] | **AN** | **15.0** | 12.0-18.0 | 0.945 (ns) |
|  | **Ctrl** | **15.0** | 12.0-18.0 |  |
| body mass  [kg] | **AN** | **37.0** | 24.0-49.0 | <0.001 |
|  | **Ctrl** | **52.0** | 35.0-75.0 |  |
| height  [cm] | **AN** | **162.0** | 136.0-180.0 | 0.037 |
|  | **Ctrl** | **165.0** | 143.0-177.0 |  |
| BMI  [kg/m^2^] | **AN** | **14.4** | 10.7-19.1 | <0.001 |
|  | **Ctrl** | **19.1** | 15.2-25.7 |  |
| IBW  [%] | **AN** | **55.7** | 38.6-94.1 | <0.001 |
|  | **Ctrl** | **73.1** | 57.1-98.5 |  |
| TTI | **AN** | **10.0** | 3.0-36.0 | - |
| PCR  [%] | **AN** | **40** | 10.0-100.0 | <0.001 |
|  | **Ctrl** | **0** | 0.0-54.0 |  |

Results are expressed as **Median** with max. and min. range. Significant values from p < 0.05, ns- statistically non-significant, n- number of patients, SD- standard deviation. Statistical tests used: Mann-Whitney U test, t- test or Welch test. BMI- Body Mass Index [kg/m^2^], IBW- % of Ideal Body Weight (fraction) TTI- total duration of illness [months], PCR- Plaque Control Record Index.

Supplementary table S2. Concentration of opiorphin, immunoglobulins IgA, IgG and IgM in unstimulated whole saliva.

| group  variable | AN  n=83 | Ctrl  n=79 | p-value |
| --- | --- | --- | --- |
| IgA  [μg/ml] | **159.2** (12.6-866.5) | **94.3** (10.3-885.8) | p=0.143 (ns) |
| IgG  [pg/ml] | **5134.5** (1025.2-12177.3) | **5839.1** (1155.7-11921.2) | p=0.344  (ns) |
| IgM  [ng/ml] | **381.3** (10.6-705.4) | **492.4** (12.0-561.0) | p<0.001 |
| opiorphin [ng/ml] | **1.1** (0.001-22.6) | **0.6** (0.005-4.7) | p<0.001 |

The results are expressed as **Median** and min-max ranges. Significant values from p≤0.05, p≤0.01, p≤0.001, ns- statistically non-significant, n- number of patients. Statistical tests used: Mann-Whitney U test, t- test or Welch test.
